# Detection of IL-17A and IL-17F gene polymorphism in recurrent and disseminated pityriasis versicolor: a case-control study

**DOI:** 10.1007/s00403-022-02462-9

**Published:** 2022-11-30

**Authors:** S. R. El-Tahlawi, A. H. Ramadan, O. G. Shaker, R. F. Hilal

**Affiliations:** 1 Department of Dermatology, Kasr Al Ainy Teaching Hospital, Faculty of Medicine, Cairo University, Al-Saray Street, Kasr Al Ainy, El Manial, Cairo, 11956 Egypt; 2 Department of Medical Biochemistry and Molecular Biology, Faculty of Medicine, Cairo University, Al-Saray Street, Kasr Al Ainy, El Manial, Cairo, 11956 Egypt

**Keywords:** Pityriasis versicolor, Gene polymorphism, IL-17A, IL-17F

## Abstract

Recurrent and disseminated pityriasis versicolor (RDPV) is a common clinical entity, characterized by its recurrent and disfiguring nature. Studies demonstrated host genetic variations in the immune response, especially the role of IL-17 in antifungal immunity. This study aimed to detect whether IL-17A and F gene polymorphisms are found in cases of RDPV. It included 100 cases of RDPV and 100 age and sex matched controls, from which EDTA blood samples were taken for single-nucleotide polymorphism analysis. IL-17A (rs2275913) and F (rs763780) were associated with a significantly increased incidence of developing RDPV. IL-17A and F gene polymorphism could be implicated as a risk factor for the development of RDPV.

## Introduction

Pityriasis versicolor (PV) is a superficial mycotic infection, commonly encountered in tropical regions due to hot, humid climates. The causative fungus belongs to the *Malassezia* genera, which constitutes part of the cutaneous microflora in humans [1].

Recurrent and disseminated PV (RDPV) is a clinical entity seen in both immunocompetent and immunosuppressed patients, previously characterized with certain features [2]. Such patients are proposed to have a genetic predilection [3] and a recalcitrant response to antifungals [4]. Defective host immunity is a pivotal factor that appears to predispose these patients to RDPV, and data regarding this field of research appears to be limited.

Interleukin 17 (IL-17), a pro-inflammatory cytokine, has established rising importance, as a culprit as well as a therapeutic target, in quite a number of diseases [5]. IL-17A is the prototypical member of the IL17 family, having the strongest impact on health and disease [6]. This importance is attributed to its key role in promoting the host defense mechanisms against bacterial and fungal infections [7]. IL-17A is secreted by a subset of T Helper (Th) cells, namely Th-17, among other cells [8]. Chemokine (C–C motif) ligand 20 (CCL20) is a chemokine which binds to its receptor Chemokine receptor 6 (CCR6), attracts Th-17 cells, and is considered a marker for Th-17 cells [9]. To fight infection, IL-17 potentiates granulopoiesis and neutrophilia through the release of granulocyte monocyte colony stimulating factor (GM-CSF) and induces neutrophil influx through the release of neutrophil chemoattractants such as IL-8, C-X-C Motif Chemokine Ligand 8 **(**CXCL8) [10]. The migration of lymphocytes, dendritic cells and monocytes was found to be also stimulated by IL-17 [9].

IL-17 family comprises six members, IL-17A to F, identified by sequence screening. IL-17A and F show 50% homology and are secreted as homodimers and heterodimers. IL-17F shares most actions with the more potent IL-17A [11, 12]. IL-17A and F are mainly produced by immune cells, while IL-17B, C and D are mainly produced by epithelial cells [13]. IL-17A and F are co-expressed on linked genes [10]. Therefore, conducting analyses of both IL-17A and F gene polymorphism could strengthen their implication in relevant pathologic conditions.

As a concise overview, it can be outlined that both IL-17A and F show strong pro-inflammatory actions through potent recruitment of immune cells, have synergistic actions with other cytokines such as tumor necrosis factor-α (TNFα-), IL-1$\beta$ and GM-CSF [14], are central to the epithelial barrier homeostasis through release of antimicrobial peptides [15], contribute to B cell stimulation [16], thus allowing the interplay between both innate and acquired immunity. A disorder in any of these immunological checkpoints may possibly contribute to the development of RDPV.

Previously published reports within the setting of fungal infections highlighted that IL-17A and 25 deficient mice have been found to show higher fungal burden after infection with *Candida Albicans*, which in turn improved by exogenous administration of IL-17A [11]. IL-17F plays an important role in inflammatory responses and protection at barrier surfaces, as shown by an intensified susceptibility to chronic mucocutaneous candidiasis in humans with an IL-17F deficiency [17].

In an attempt to further clarify how a defective host immunity might contribute to the development of RDPV, which is considered as a clinical dilemma; and whether IL-17, especially the most relevant IL-17A and F, might play a role within this context, this study was formulated.

## Patients and methods

This observational case–control study included 100 patients with RDPV and 100 age and sex matched healthy individuals who served as controls and were not relatives of the patients. All were recruited from the dermatology outpatient clinic, Cairo University Hospital. The study was approved by the Research Ethics Committee, Faculty of Medicine, Cairo University. This study was conducted during the period from October 2020 until October 2021.

**Fig. 1:**
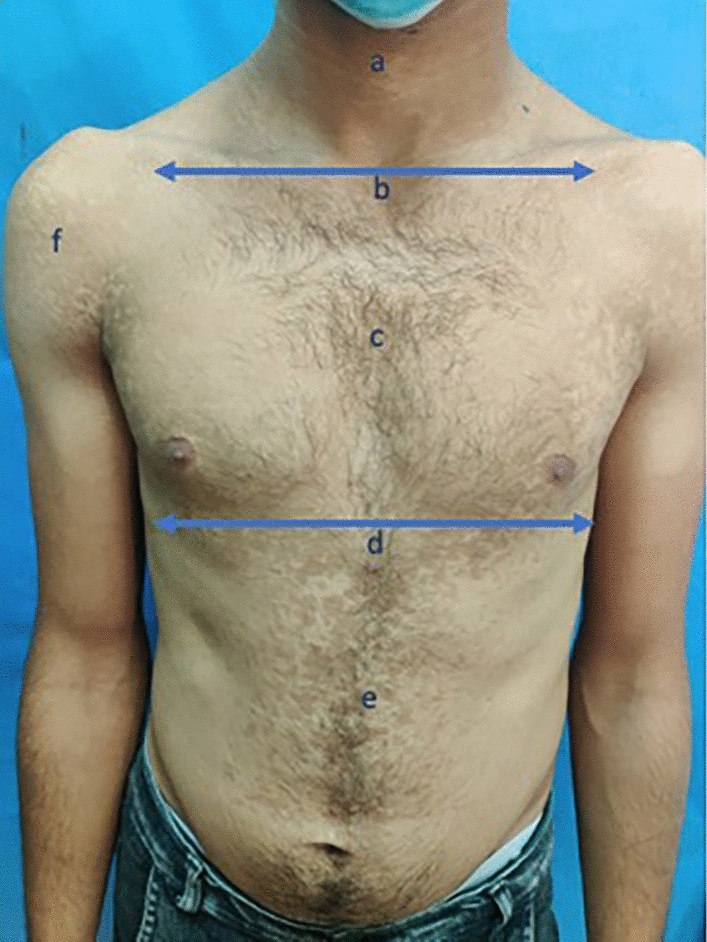
Skin mapping for determination of area affection as follows; **a** Neck affection, **b** Infra-clavicular line, **c** Chest affection, **d** Infra-mammary line, **e** Abdomen affection and **f** Upper limb affection

**Fig. 2:**
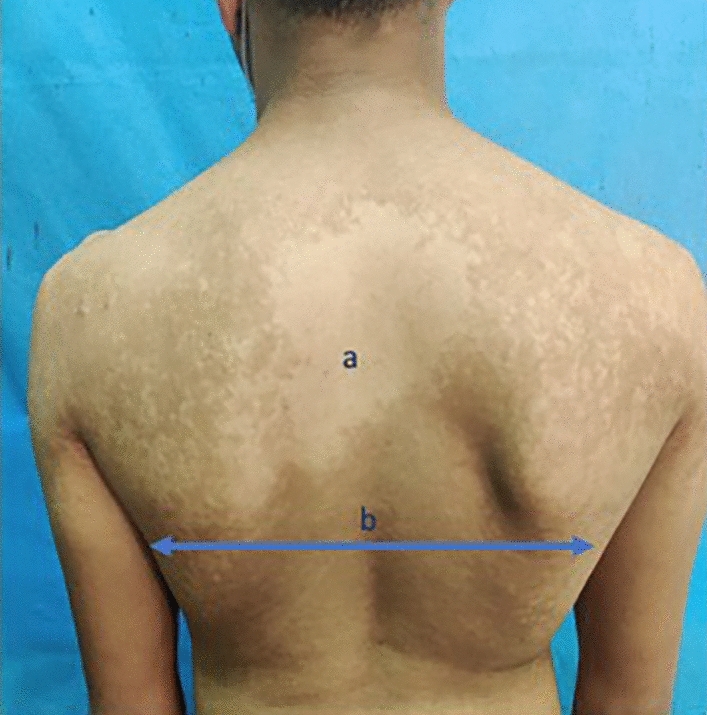
Skin mapping for determination of area affection as follows; **a** Upper chest affection and **b** Infra-scapular line

Patients who were over 18 years of age, of both genders and fit the criteria of active RDPV were included in the study. Active RDPV patients documented 2 or more episodes per year, showed both a positive Wood’s light and direct mycological examination for Malassezia species using Potassium hydroxide (KOH) direct microscopy, presented with lesions that involved two or more contiguous anatomical areas [face and neck (as one area, above infra-clavicular line), chest (above infra-mammary line), abdomen (below infra-mammary line), upper back (above infra-scapular line), lower back (below infra-scapular line) and upper limbs], (Figs. 1 and 2).

Patients with other cutaneous diseases, autoimmune diseases (e.g., Systemic Lupus Erythematosus, Dermatomyositis, etc.), immunosuppression (e.g., HIV patients, organ transplants, immunosuppressive drugs) were excluded from the study. Pregnant and lactating females were also excluded.

An informed written consent for participation, photography and publication was signed by all patients eligible for participation.

## Patient assessment

All patients were subjected to thorough history taking including age, gender, skin type, comorbidities, onset, course, duration of the disease, disease activity, number of previous episodes, previous therapeutic attempts, as well as history of other skin disorders, family history of pityriasis versicolor or other skin disorders.

All controls were subjected to thorough history taking including age, gender, skin type, comorbidities, history of any skin diseases especially those with skin pigmentation and family history of pityriasis versicolor.

Skin examination was performed under daylight as well as under Wood’s light for a better observation of clinical changes, to confirm the diagnosis. Total body skin examination was done to exclude any other cutaneous diseases that could prevent the subject from being enrolled in the study.

**Fig. 3:**
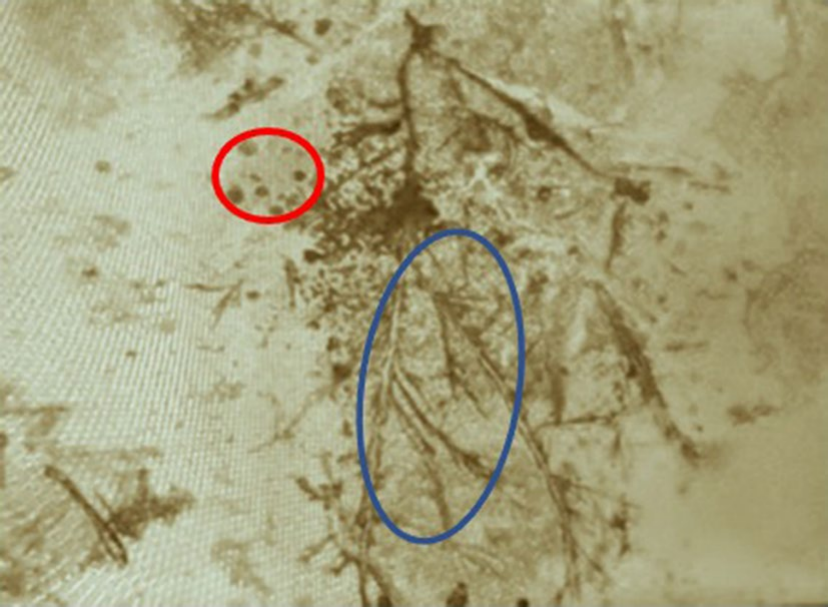
KOH examination showing spaghetti and meatball appearance. Blue outline encircles spaghetti (hyphae), Red outline encircles meatballs (spores). Magnification X10

To confirm the clinical diagnosis in all patients, KOH and direct microscopy were done using skin scrapings. Detection of “spaghetti and meat ball” appearance was considered confirmatory, (Fig. 3).

## Blood sampling

From each participant, 3 ml of venous blood was withdrawn under complete aseptic conditions and put in EDTA tubes for DNA extraction of IL-17A and F genotyping.

Samples in EDTA tubes were either stored in the same vacutainer at -20 °C or used directly within 24 h of blood withdrawal for DNA extraction using the extraction kit.

Detection of single-nucleotide polymorphism (SNP) was performed using real-time PCR (polymerase chain reaction) with specific primers and probe.

SNP was examined for both IL-17A and F, respectively, using the TaqMan technique.

**Fig. 4:**
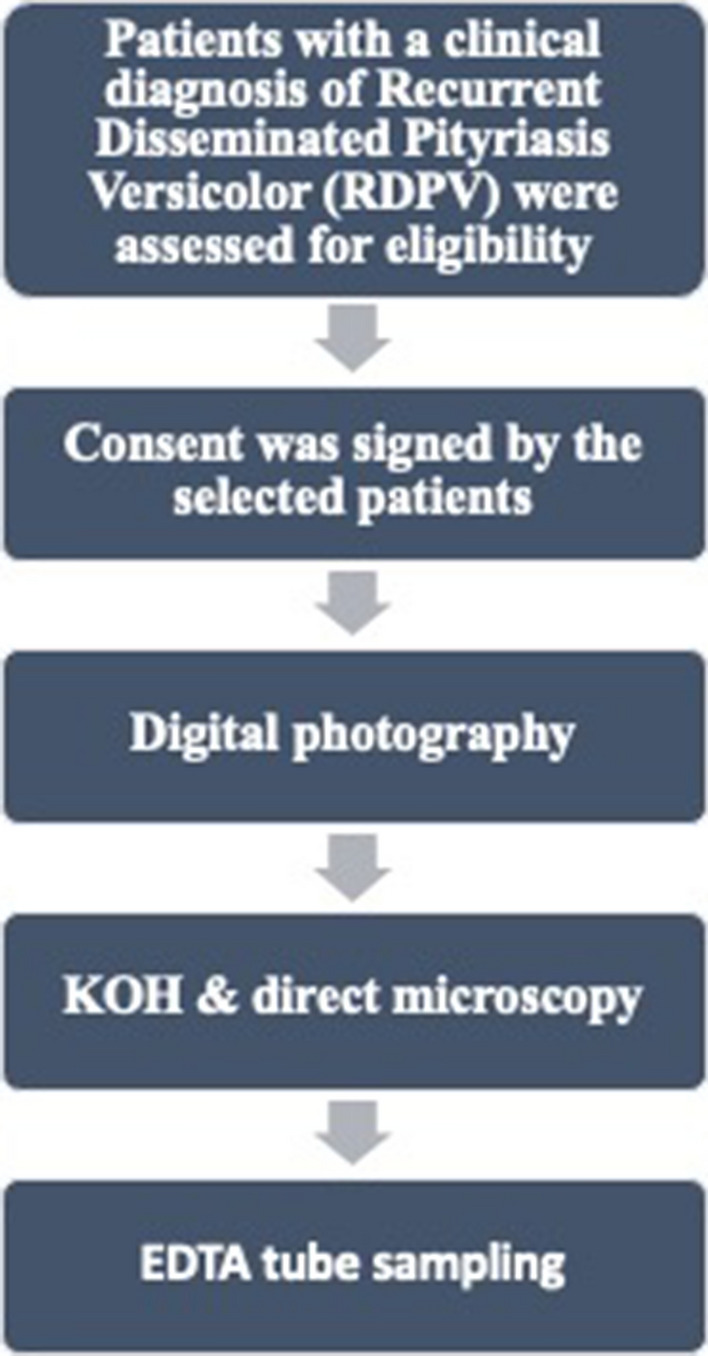
Flowchart showing patient recruitment and evaluation scheme

A flow diagram illustrates a stepwise evaluation scheme for the cases’ enrollment plan (Fig. 4).

## Assessment of genetic polymorphisms of rs2275913 guanine/adenine (G/A) and rs763780 thymine/cytosine (T/C)

These SNPs were transition substitution types. DNA was extracted from EDTA blood using QIAamp kit supplied by Qiagen (USA, catalogue number 51306) according to manufacturer's specifications. Predesigned primer/probe sets for the 2 genotypes were used (Applied Biosystems, USA). Probes were synthesized with reporter dye FAM or VIC covalently linked at the 5/ and a quencher dye MGB linked to the 3/ end of the probe (ThermoFisher Scientific, USA). DNA was amplified using TaqPCR Master Mix Kit supplied by Qiagen (catalogue number 201443). A reaction mix was prepared as follows: 12.5 µl Taqman master mix., 1.25 µl primer/probe (SNP ID; rs2275913and SNP ID; rs763780) and 1 µl DNA (~ 100 ng) in a total volume of 25 µl.

Real-time PCR was performed using a Rotor gene Q Real-Time PCR System (Qiagen, Valencia, CA, USA) with the following conditions: One cycle for 10 min at 95 $\mathrm{^\circ{\rm C} }$ followed by 45 cycles of 95 $\mathrm{^\circ{\rm C} }$ for 30 s and 60 $\mathrm{^\circ{\rm C} }$ for 1 min., and fluorescence was measured at the end of every cycle and at the endpoint.

## Statistical methods

The data collected were coded and tabulated in Microsoft Excel worksheet and computerized analysis was done utilizing computer program IBM SPSS (Statistical Package for the Social Science; IBM Corp, Armonk, NY, USA) release 26 for Microsoft Windows.

A descriptive statistical analysis was carried out for presenting the parameters under study. Data were summarized using mean and standard deviation for quantitative variables and frequencies (number of cases) and relative frequencies (percentages) for categorical variables. Comparisons between quantitative variables were done using the nonparametric Kruskal–Wallis and Mann–Whitney tests. For comparing categorical data, Chi square (χ2) test was performed. Exact test was used instead when the expected frequency is less than 5. Genotype and allele frequencies were compared between the disease and the control groups using logistic regression. Odds ratio (OR) with 95% confidence intervals was calculated. P-values less than 0.05 were considered as statistically significant [18–20].

## Results

The demographic data of both cases and controls showed no significant difference with regard to the age and gender, as presented in Table 1.

**Table 1:** Demographic data of patients and controls

	RDPV			Controls			*P* value
	Mean ± SD	Median	Range	Mean ± SD	Median	Range	
Age	30.19 ± 10.83	28.00	18.00–72.00	31.93 ± 10.39	30.00	18.00–70.00	0.15

Gender	Count (%)		Count (%)		
	M	F	M	F	0.19
	67 (67)	33 (33)	58 (58)	42 (42)	

Nonparametric Mann–Whitney test for quantitative data, Chi square (χ2) test for qualitative data, *P* value significant < 0.05

*RDPV* recurrent and disseminated pityriasis versicolor, *M* male, *F* female, % percentage, *SD* standard deviation

Family history of RDPV was found positive in 40 patients (40%) and 16 controls (16%), with a highly significant difference between both (*P* < 0.001). The patients with 3 area affection were significantly greater in number than those with 2 or 4 area affection (*P* < 0.001). Baseline clinical data are summarized in Table 2.

**Table 2:** Clinical data of the patients including duration since the 1^st^ episode of pityriasis versicolor, the number of following relapses per year and the number of areas affected per patient

	Mean ± SD	Median	Range
Duration (years)	5.04 ± 4.43	3	1–25
Number of relapses (/year)	4.96 ± 4.46	3	2–20

		Count (%)	*P* value
Number of areas affected	2	21 (21)	Overall *P* value: < 0.001 2 area vs 3 area affection: < 0.001 2 area vs 4 area affection: 0.39 3 area vs 4 area affection: 0.005
	3	52 (52)	
	4	27 (27)	

Kruskal–Wallis test, Mann–Whitney as post hoc test, *P* values < 0.05 statistically significant

% percentage, *SD* Standard deviation

### IL-17A gene polymorphism data

During laboratory processing of IL-17A gene polymorphism analysis, DNA was lost in one patient sample, with a resultant of a total of 99 patient samples and 100 control samples.

IL-17A rs2275913 (G/A) gene polymorphism was used, where the wild (normal) allele was G and the mutant allele was A. The homozygous normal gene was GG, which was used as a reference. The homozygous mutant gene AA and the heterozygous gene GA were examined in both the patients and the control groups.

The homozygous mutant gene (rs2275913) AA of IL-17A was found in 55 (55.6%) patients, the heterozygous mutant gene GA was found in 38 (38.4%) patients and only 6 (6%) patients had the wild (normal) gene GG. The homozygous mutant gene (rs2275913) AA of IL-17A was found in 20 (20%) controls, the heterozygous mutant gene GA was found in 37 (37%) and 43 (43%) controls had the wild (normal) gene GG, (Table 3).

**Table 3:** Comparison of homozygous, heterozygous and combined homo- and heterozygous IL-17A mutant gene vs wild (normal) gene in patients and control

		RDPV	Controls	*P* value	OR	95% CI	
		Count (%)	Count (%)			Lower	Upper
rs2275913 (G/A)	AA	55 (55.6)	20 (20)	< 0.001	19.71	7.28	53.35
	GA	38 (38.4)	37 (37)	< 0.001	7.36	2.80	19.35
	AA + GA	93 (94)	57 (57)	< 0.001	11.69	4.68	29.21
	GG	6 (6)	43 (43)	Reference			

Logistic regression model, *P* values < 0.05 statistically significant

*RDPV* recurrent and disseminated pityriasis versicolor, *OR* odds ratio, *CI* confidence interval, % percentage, *A* adenine, *G* Guanine

The homozygous mutant gene (rs2275913) AA of IL-17A polymorphism was associated with a 19.708-fold (*P* < 0.001) increase in susceptibility to RDPV infection in the patient group. Similarly, the heterozygous mutant gene (rs2275913) GA of IL-17A polymorphism was associated with a 7.360-fold (*P* < 0.001) increase in susceptibility to RDPV infection in the patient group. Combination of both homozygous and heterozygous IL-17A (rs2275913) AA and GA was associated with 11.693-fold (*P* < 0.001) increase in susceptibility to RDPV infection in the patient group, (Table 3).

Analysis of the mutant allele (A) and (G) data was performed in comparison with the total number of alleles which was 198 patient alleles, and 200 control alleles, since each sample possesses 2 alleles.

The allelic frequencies were detected as follows; mutant allele (A) counted as 148 (74.7%) patient alleles and 77 (38.5%) control alleles, while the wild (normal) allele (G) counted as 50 (25.3%) patient alleles and 123 (61.5%) control alleles. A comparison between the mutant allele (A) versus the wild (normal) allele (G) revealed a 4.728-fold (*P* < 0.001) increase in susceptibility to RDPV infection in the patient group.

### IL-17F gene polymorphism data

No DNA was lost during laboratory processing of IL-17F gene polymorphism analysis, with a total of 100 patient samples and 100 control samples.

IL-17F rs763780 (T/C) gene polymorphism was used, where the wild (normal) allele was T and the mutant allele was C. The homozygous normal gene was TT, which was used as a reference. The homozygous mutant gene CC and the heterozygous gene TC were examined in both the patients and the control groups.

The homozygous mutant gene (rs2275913) CC of IL-17F was found in 3 (3%) patients, the heterozygous mutant gene TC was found in 81 (81%) patients and 16 (16%) patients had the wild (normal) gene TT. The homozygous mutant gene (rs2275913) CC of IL-17F was not found in controls, while the heterozygous mutant gene TC was found in 36 (36%) and 64 (64%) controls had the wild (normal) gene GG, (Table 4).

**Table 4:** Comparison of homozygous, heterozygous and combined homo- and heterozygous IL-17F mutant gene vs wild (normal) gene in patients and control

		RDPV	Control	*P* value	OR	95% CI	
		Count (%)	Count (%)			Lower	Upper
rs763780 (T/C)	CC	3 (3)	0 (0)	0.999	–	–	–
	TC	81(81)	36 (36)	< 0.001	9	4.59	17.66
	CC + TC	84 (84)	36 (36)	< 0.001	9.33	4.76	18.29
	TT	16 (16)	64 (64)	Reference			

Logistic regression model, *P* values < 0.05 statistically significant

*RDPV* recurrent and disseminated pityriasis versicolor, *OR* odds ratio, *CI* confidence interval, % percentage, *T* thymine, *C* cytosine

The homozygous mutant gene (rs2275913) CC of IL-17F polymorphism was not associated with an increase in susceptibility to RDPV infection in the patient group. On the contrary, the heterozygous mutant gene (rs2275913) TC of IL-17F polymorphism was associated with a ninefold (*P* < 0.001) increase in susceptibility to RDPV infection in the patient group. Combination of both homozygous and heterozygous IL-17F (rs2275913) CC and TC was associated with 9.333-fold (*P* < 0.001) increase in susceptibility to RDPV infection in the patient group, (Table 4).

Analysis of the mutant allele (C) and (T) data was performed in comparison with the total number of alleles which was 200 patient alleles, and 200 control alleles, since each sample possesses 2 alleles.

The allelic frequencies were detected as follows; mutant allele (C) counted as 87 (43.5%) patient alleles and 36 (18%) control alleles, while the wild (normal) allele (T) counted as 113 (56.5%) patient alleles and 164 (82%) control alleles. A comparison between the mutant allele (C) versus the wild (normal) allele (T) revealed a 3.507-fold (*P* < 0.001) increase in susceptibility to RDPV infection in the patient group.

## Discussion

The recurrent and cosmetically disfiguring nature of recurrent and disseminated pityriasis versicolor (RDPV) appears to stigmatize the quality of life of such patients, who do appear healthy and do not manifest any remarkable features of immune suppression. Most patients presenting with classic PV show a satisfactory response to antifungal treatment, except for those who evolve into RDPV and thus, present with a highly recurrent course and somewhat disappointing results to treatment [4]. For this reason, this study aimed to address a possible underlying genetic defect, and so included only patients with RDPV. Patients with IL-17A rs2275913 (G/A) and to a lesser extent IL17 F rs763780 (T/C) gene polymorphism showed a significantly higher incidence (11.693-fold, 9.333-fold, respectively) of developing RDPV. Furthermore, a homozygous mutation of IL-17A is implicated with higher incidence and more susceptibility to the disease. This, together with the co-implication of both IL-17A and F polymorphism, suggest a strong evidence of a genetic defect related to the condition of RDPV. The genetic etiology of RDPV is further supported by the finding of a statistically significant difference between patients and controls as regard to the family history, (*P* < 0.001).

To date, this is the first study to detect a possible association between mutant IL-17A (rs2275913), mutant IL-17F (rs2275913) genes and the patient’s susceptibility to RDPV.

An experimental model used to study the host response to Malassezia in the skin in vivo revealed the role of IL-17 in antifungal immunity, whereby IL-17 deficiency resulted in impaired fungal control [21].

A correlation was found between IL-23/IL-17 gene polymorphisms in a Chinese population and candida infection (vulvovaginal candidiasis), and this study concluded that IL-17 gene polymorphism increases the risk of vulvovaginal candidiasis [22]. Within the same context, another study suggested that gene polymorphism in Toll like receptor 2 reduces the production of IL-17 and thereby increases susceptibility to recurrent vulvovaginal candidiasis [23].

On a different note, a study came to the conclusion that blockage of the IL-17 signaling pathway increased the incidence of superficial fungal infections (including three cases with pityriasis versicolor) in psoriatic patients [24]. Rapid onset of widespread tinea versicolor has been encountered in a psoriatic patient following a switch from ustekinumab to ixekizumab [25]. Another psoriatic patient on secukinumab was reported to present with atypical oral candidiasis, which improved remarkably following lowering the monthly dose of secukinumab to 150 mg [26] as well as another case of psoriasis who experienced co-existing chronic hyperplastic candidosis and an oral lichenoid lesion, as  side effects of the secukinumab therapy, which the patient was subjected to [27]. These reports emphasize the importance of IL-17 in the pathogenesis of PV as well as other superficial fungal infections.

Cytokine gene polymorphism analyses play a pivotal role in exploring possible etiologic factors of morbidities. Such morbidities may exhibit inconclusive pathogeneses and inconsistent response to treatments, as is the case with RDPV, thereby adding value to gene polymorphism studies.

RDPV is a clinical entity characterized by a chronic, remitting course, socially stigmatizing the patients, with a negative impact on the patients’ quality of life [2]. Antifungal resistance has been questioned as a cause for the recalcitrant nature of the disease, yet published studies confirmed high antifungal activity of itraconazole, voriconazole and ketoconazole against *Malassezia sp*. [28]. Although RDPV can be a manifestation of immune suppression, RDPV has been encountered in immunocompetent individuals [2], suggesting that both an overt as well as a subtle cell mediated immune disturbance can cause the condition [29]. A Th2 polarized immune axis is also proposed to be an important cornerstone in the occurrence of RDPV [30].

RDPV was described in a previous study including 16 patients, which proclaimed the term as having more than three non-contiguous affected areas, with lesions of more than 10 cm in at least one of its extensions, and two or more episodes of PV in 1 year [2].

The parameters in the current study of one hundred PV patients, however, show that the term RDPV should be re-defined to having ≥ 2 relapses of pityriasis versicolor per year in an immunocompetent individual, having at least 2 contiguous affected areas, outlined as face and neck, chest, abdomen, upper limbs, upper and lower back. Such criteria allow an easy more straightforward clinical profiling of the patients.

It is anticipated that this study highlighted that IL-17 A and F may play a role in RDPV pathogenesis and provided a proposed explanation for the appearance of this condition in otherwise healthy patients. Re-categorization of RDPV clinical features has also been described by this study. Furthermore, the results of this study can pave the way for the future emergence of novel therapeutic agents such as IL-17 therapy, as in the animal study that reported reversal of the fungal infections on exogenous administration of IL-17 [11], or introduction of treatments that potentiate the action of IL-17, in various chronic fungal conditions including RDPV.

Limitations of this study include that IL-17 levels were not measured. A genetic etiology would have been more strongly evidenced if a comparison with classic PV patients was included, although previous gene polymorphism studies done on diseases with a recurrent nature were conducted with healthy controls [23, 31].

RDPV is a common, cosmetically disfiguring condition that seems quite challenging to clinical practitioners and annoying to patients. As a conclusion, it is proposed to consider RDPV as a separate entity that genetically and immunologically differs from the sporadic self-limited form of PV. Aiming to find potential causal factors, IL-17A and IL-17F gene polymorphisms have been found to be probably implicated in the pathogenesis of RDPV. Large-scale genetic studies to detect other genes affecting the incidence, severity, recurrence, or course of the disease are recommended.

## Data Availability

The data supporting findings of this study are available within the article. Raw data from which the findings of this study were obtained, are available from the corresponding author, upon request.
